# The performance of body mass component indices in detecting risk of musculoskeletal injuries in physically active young men and women

**DOI:** 10.7717/peerj.12745

**Published:** 2022-01-26

**Authors:** Jarosław Domaradzki, Dawid Koźlenia

**Affiliations:** Physical Education and Sport, Wroclaw University of Health and Sport Sciences, Wroclaw, Dolnośląskie, Poland

**Keywords:** Body composition indices, Musculoskeletal injuries, Physical activity, Young adults, Body mass index (BMI), Fat mass index (FMI), Relative muscle mass (SMI), Muscle to body fat ratio (MFR)

## Abstract

**Background:**

Body composition indices can be related to musculoskeletal injuries (MI), particularly in physically active groups. However, little is known about the accuracy of these diagnoses as potential predictors of musculoskeletal injuries. Therefore, this study aims to indicate the cut-off points of relative body mass (BMI), fat mass index (FMI), skeletal muscle mass (SMI), and muscle to fat ratio (MFR) and establish its reliability in injury prediction for physically active men and women.

**Methods:**

The sample included 119 physically active individuals aged 23.72 ± 1.12 (66 men body height 1.79 ± 0.07 (m); body weight 80.51 ± 9.85 (kg) and 53 women body height 1.67 ± 0.08 (m); body weight 62 ± 10.72 (kg)), students at university of physical education in Poland. The participants’ physical activity was measured with the International Physical Activity Questionnaire (IPAQ). The relevance of body mass index (BMI), fat mass index (FMI), skeletal muscle mass index (SMI), and muscle to fat ratio (MFR) in detecting injury risk was examined. Musculoskeletal injuries during 1 year before examination were registered using a self-reporting questionnaire. The areas under the curve (AUC) and Youden Index in the receiver operating characteristic curve (ROC) were calculated.

**Results:**

The cut-off points used to classify the indices among men were BMI = 24.38; FMI = 3.74; SMI = 16.40; MFR = 3.70; and for women BMI = 20.90; FMI = 4.17; SMI = 8.96; MFR = 1.67. Results suggested the greatest reliability in the prediction of musculoskeletal injuries among men had BMI (AUC = 0.623; Youden = 0.30) and SMI (AUC = 0.608; Youden = 0.23) whereas among women, MFR (AUC = 0.628; Youden = 0.29) and FMI (AUC = 0.614; Youden = 0.32).

**Conclusion:**

BMI and SMI are the most appropriate indices to predict the risk of musculoskeletal injury in physically active men, whereas in women, MFR and FMI are more reliable. These results indicate that the indices with more muscle mass meaning are better in predicting injury among men. In contrast, indices with a higher contribution of fat are better for women. It indicates sex differentiation of injury risk conditions. Men should focus on developing muscle mass, whereas women should reduce body fat to decrease injury risk. However, widespread use seemed to be limited to the specific examined group. Therefore, cut-off points should be used with caution, and calculated values should be verified and confirmed in subsequent studies.

## Introduction

Being physically active is a critical behavior habit for being healthy. Numerous studies have documented the many benefits of a physically active lifestyle for health and well-being (Brown et al., 2004; Santos, 2013; Vuillemin et al., 2005). But, physical activity has not only a positive influence on health. It also has negative aspects, such as musculoskeletal injuries (Złotkowska et al., 2015). There are some factors associated with increased injury risk during physical activity.

The risk factors for MI are classified as extrinsic (*e.g*., inadequate effort load, inappropriate equipment, and weather conditions) and intrinsic (*e.g*., sex, age, flexibility, physical performance, high body fat, and low skeletal muscle mass) (Havenetidis et al., 2017; Melloni et al., 2018). Both types of risk factors are reasons for the increasing prevalence of injuries in different populations (Taanila et al., 2015). Articles presenting systematic studies showed a range of injury incidents among various types and level physically active subjects between 51–70% (Taanila et al., 2010; Murphy, Connolly & Beynnon, 2003). Sieńko-Awierianów & Chudecka (2020) found that 40% of physically active athletes reported MI related to physical training in Poland. Moreover, men (45.71%) more often than women (35.51%) had a history of injuries. The most common circumstances were running or jumping on uneven ground. However, the site of physical activity seems to be irrelevant. Participants of both outdoor and indoor physical activity are exposed to MI. Other studies show the prevalence of injuries among people in sports centers. Respondents (41.6%) reported an injury from exercising on gym equipment (Lubetzky-Vilnai, Carmeli & Katz-Leurer, 2009).

Most of the studies mentioned above are related to military or athlete populations—relatively few document MI in healthy, physically active young adults. However, professional athletes and military personnel are under a specific training program for physical performance improvement in opposition to the average physically active population. Therefore, injury risk should interpret accordingly. Moreover, there are no studies of the intrinsic factors related to body dimensions or body composition. Some studies show simple relationships between body height, weight, and BMI with injury (Mehri et al., 2010; Santos et al., 2014; Hollander et al., 2020). Therefore, it seems that if there were known values of various body composition indices related to the high risk of MI, preventing such injuries would be more effective.

The risk of injury depends on the individual characteristics of an athlete (intrinsic) and environmental (extrinsic) risk factors. The first group is biological factors such as body size (body height and weight), leg length, and anthropological asymmetries (Zaar et al., 2017). A literature review indicated only a few articles examined body composition various indices as injury risk factors (Nye et al., 2018; Taanila et al., 2010). In only one study, authors tried to identify critical values of composition indices related to increased injury risk (Havenetidis et al., 2017). Cut-off points were indicated for body mass index (BMI), free fat mass index (FFMI), and body fat percentage (BFP).

All the studies mentioned above were designed mainly for populations of military trainees and conscripts, and professional sports competitors (Santos, 2013; Melloni et al., 2018; Mehri et al., 2010; Taanila et al., 2010). Healthy, physically active young adult populations have not yet been sufficiently described in studies that examine cut-off points of body composition indices for high (or low) risk of the most common injuries. Identifying intrinsic risk factors is essential to preventing injuries instead of treating them. More physical activity-induced injuries among young adults are severe problems in public health care (Złotkowska et al., 2015).

To the best of the authors’ knowledge, no studies have examined cut-off points for skeletal muscle mass index (SMI) and proportions between muscle mass to fat index (MFR). Moreover, there are no studies conducted on physically active young men and women. Thus, our investigation was focused on identifying the values of various body composition indices which can predict the increased risk of injury in a healthy, average population. Therefore, this study aims to establish the cut-off points of relative body mass (BMI), fat mass index (FMI), skeletal muscle mass (SMI), and muscle to fat ratio (MFR) and establish its reliability in injury prediction for physically active men and women. Therefore, we hypothesized that the body composition indices mentioned above would provide a useful predictive tool in predicting MI in physically active young adult populations.

## Materials and Methods

### Participants

Initially, 138 subjects were to be included in this research. However, due to incomplete measurements or not meeting inclusion criteria, 19 subjects were excluded. Finally, the study examined 119 young, physically active students, 66 men aged 23.94 ± 1.18, physical activity level 3,504.454 ± 2,311.09 (MET) and 53 women aged 23.43 ± 0.97 physical activity level 4,178.57 ± 2,626.54. A detailed description of the study sample is included in Table 1. The level of physical activity was measured with the International Physical Activity Questionnaire (IPAQ); Polish version by Biernat, Stupnicki & Gajewski (2007), and was an estate on sufficient level as include factor (3 or more days of intense physical activity not less than 20 min a day = 480 METs/week, or 5 or more days of moderate exercise or walking not less than 30 min per day = 600 METs/week, or 5 or more days of any combination of physical activity (walking, moderate or vigorous exercise) exceeding 600 METs/week). The participants declared having no experience in professional sports and not suffering any injuries 6 weeks before the measurements. The measurements were taken during the morning hours. Only participants practicing for at least PA 5 years were included in studies. We intended to ensure the high homogeneity of the group concerning the risk of injuries. The IPAQ instrument is open access, and no permissions are required for its use (The IPAQ Group, 1999).

**Table 1:** Descriptive statistics of the outcomes in the whole group and each sex group.

Group	whole	men	women
Variable	mean (±sd) 95% CI	mean (±sd) 95% CI	mean (±sd) 95% CI
body height (cm)	1.74 (0.1) [1.73–1.76]	1.79 (0.07) [1.78–1.82]	1.67 (0.08) [1.65–1.69]
body weight (kg)	72.27 (13.77) [69.77–74.76]	80.51 (9.85) [78.09–82.93]	62 (10.72) [59.04–64.95]
BMI (kg/m^2^)	23.65(3.13) [23.38–24.22]	24.89 (2.58) [24.26–25.52]	22.11 (3.09) [21.25–22.96]
FMI (kg/m^2^)	4.94 (1.66) [4.63–5.24]	4.53 (1.34) [4.2–4.86]	5.45 (1.88) [4.93–5.97]
SMI (kg/m^2^)	13.13 (3.94) [12.41–13.84]	15.72 (2.97) [14.99–16.45]	9.89 (2.23) [9.28–10.51]
MFR (kg/kg)	2.9 (1.21) [2.68–3.12]	3.69 (1.05) [3.43–3.95]	1.93 (0.46) [1.8–2.05]
body fat (kg)	14.95 (5.07) [14.03–15.87]	14.62 (4.30) [13.56–15.68]	15.37 (5.90) [13.74–16.99]
body muscles (kg)	40.84 (15.47) [38.03–43.65]	51.2 (11.88) [48.28–54.12]	27.95 (7.82) [25.8–30.10]

### Anthropometry

Height (0.1 cm) and weight (0.1 kg) were measured by experienced staff (more than 100 various measures per year) from the Department of Biostructure of the Wroclaw University of Physical Education. The instrument was a height gauge model 764 (SECA manufactured, Hamburg, Germany, with quality control number C-2070). The measurements were conducted following the anthropometric measurement standards delineated by the International Society for the Advancement of Kinanthropometry (ISAK). Based on the values of height and weight obtained, the index of relative body mass–BMI (kg/m^2^)–was calculated using the following formula:

${\rm BMI} = {\displaystyle{{{\rm body\; mass\; }\left( {{\rm kg}} \right)} \over {{\rm body\; height\; (}{{\rm m}^{\rm 2}})}}^{}}$

### Body composition

Bioelectrical impedance analysis (BIA) is an inexpensive and non-invasive method for assessing body composition (Pietrobelli et al., 2004). It is a simple method used in screening field tests (Yamada et al., 2013). A body monitor, TANITA MC 180 MA (Tanita Corporation, 2005), set at a 50-kHz current frequency, was used to obtain BF, MM, and free fat mass (FFM) measurements in the whole body. The distribution of BF and muscle mass (MM) was estimated using segmental bioelectrical impedance analysis (SBIA). Throughout the measurements, standardized conditions for bio-impedance measurement were maintained (Kyle et al., 2004). The subjects were asked not to undertake any physical activity, drink and eat anything at least 3 hours before the measures, and empty the bladder immediately before the measurement.

The analysis took into account the following elements of body composition: fat mass and muscle mass absolute values (kg).

Based on the body components (absolute values), the following indices were calculated. The fat mass index (FMI) was calculated from a formula like BMI:

${\rm FMI} = {\displaystyle{{{\rm body\; fat\; mass\; }\left( {{\rm kg}} \right)} \over {{\rm body\; height\; (}{{\rm m}^{\rm 2}})}}^{}}$

Relative muscle mass (height adjusted muscle mass–SMI) was calculated as follows:

${\rm SMI} = {\displaystyle{{{\rm body\; skeletal\; muscle\; mass\; }\left( {{\rm kg}} \right)} \over {{\rm body\; height\; (}{{\rm m}^{\rm 2}})}}^{}}$

Muscle to body fat ratio (MFR) was calculated as follows:

${\rm MFR} = {\displaystyle{{{\rm body\; skeletal\; muscle\; mass\; }\left( {{\rm kg}} \right)} \over {{\rm body\; fat\; mass\; (kg)}}}^{}}$

### Recording injuries

Only musculoskeletal injuries (muscle, tendon, bone, joint, or ligament injuries) were recorded and used to calculate injury prevalence. The injury occurrence was defined based on Widuchowski & Widuchowski (2005). It was described as complaints during physical activity, which resulted in pain and discomfort in the locomotor system, causing temporary limitations or a complete inability to continue physical activity.

Injuries of the sample group were collected using Injury History Survey (IHQ), a questionnaire about the injuries to the locomotor system during physical activity. The survey was supervised. Thus, the researcher was always at the disposal of the respondents (Koźlenia & Domaradzki, 2021).

### Study design

The participants were asked to complete a first questionnaire survey with the IPAQ and IHQ questionnaires on injuries sustained in the last 12 months (retrospectively). Then anthropometry and body composition measurements were conducted. The reliability of the IHQ questionnaire was verified after 7 days. The group of randomly selected 60 respondents was asked to take part in the repeated survey. Finally, the IHQ was repeated among 56 people (28 men and 28 women).

### Statistical analysis

Cronbach’s alpha coefficient was calculated to determine the reliability of the IHQ survey. It is considered that the value of the coefficient for a reliable survey tool should be at least 0.60 (Aron, Aron & Coups, 2012). The sampling frame was a list of respondents by surname in alphabetical order in the random selection for reliability test. Cronbach’s alpha coefficient was generated using the Real Statistics Resource Pack software (Release 7.6) (https://www.real-statistics.com/; Copyright (2013–2021) Charles Zaiontz).

The Shapiro–Wilk test evaluated the normality of data distribution, and all variables showed a normal distribution. Descriptive statistics of anthropometric features and body composition indices were presented as means, standard deviations, and 95% confidence intervals (CI).

The number of injuries was presented as a percentage. The chi-squared test was conducted to assess the association between sex and injuries.

The accuracy of body composition measures (BMI, FMI, SMI, and MFR) to discriminate injured from non-injured participants was assessed using the Receiver Operating Characteristic (ROC) method. The outcomes were presented visually as ROC plots of sensitivity (the proportion of true positives correctly identified by the test) compared to specificity (the ratio of true negatives accurately determined by the test) at variable cut-off points. According to analysed body composition indices, cut-off points are the optimal value for the lowest injury risk. Results further from the calculated cut-off point indicate an increased risk of injury. In addition, the area under the curve (AUC), which measures the goodness of fit and validity of the model based on sensitivity and specificity, and the standard error (SE) were calculated. These values describe the ability of the test to detect the examined characteristic (sensitivity) or to detect its absence (specificity). The Youden index was calculated from the formula:

${\rm J = maximum\; \{ sensitivity\; + \; specificity\; - \; 1\} ,\; over\; all\; cut - points\; c,\; - }\infty {\rm \; < \; c\; < }\infty {\rm .}{{\rm \; }^{}}$

The Youden index allows, based on the ROC Curve sensitivity and specificity values, to determine the optimal cut-off point (Domaradzki et al., 2021).

A *p*-value < 0.05 was considered statistically significant. The calculations were carried out using Statistica 13.0 (StatSoft Poland 2018, Cracow, Poland).

### Ethics statement

The Senate Research Ethics Committee approved Wroclaw University Health and Sport Science following institutional ethical requirements for human experiments under the Helsinki Declaration, consent number 16/2018. All subjects were required to sign a written consent before participating in this study. In addition, they were informed in detail about the purpose, type, and method of conducting the research and participation conditions. The surveys were conducted by the faculty and staff of the University of Physical Education in Wrocław.

## Results

The IHQ Cronbach’s alpha coefficient was 0.836. It indicates the high reliability and repeatability of the user survey (Aron, Aron & Coups, 2012).

Descriptive statistics of the outcomes and calculated *p*-values together with *t*-values of the independent Student’s t-test for comparison between sexes are presented in Table 1.

### Baseline characteristics

In Table 1. Descriptive statistics of body composition indices for whole study sample and regards men and women. The chi-squared test results indicated a lack of significant differences between the sexes in the number of injuries. Out of 66 men, 22 (33.33%) suffered from musculoskeletal injuries. Out of 53 women, 12 (22.64%) had experienced at least one injury during the 1 year before the tests. The rest of the participants of both sexes (men = 44, 66.67%; women = 41, 77.36%) did not suffer any musculoskeletal injury. However, these associations were not statistically significant (*χ*^2^ Yates = 1.16, *p* = 0.2805; *φ* = −0.11).

Although there was no statistical significance of the designed models, the predictive potential of all indices (except MFR in men) for identifying participants the most at risk of MI was higher than would be expected by chance (AUC > 0.5) (Table 2). Sex differences were observed in the performance of body mass component indices in detecting the risk of MI in men and women.

**Table 2:** Areas under the curve (AUC) and respective cut-off points (Youden) across all body composition indices.

Group	Index	Cut-off point	Youden index	AUC	SE	95% CI	*p*
Men	BMI	24.38	0.30	0.623	0.072	[0.483–0.764]	0.0854
	FMI	3.74	0.20	0.572	0.072	[0.430–0.714]	0.3219
	SMI	16.40	0.23	0.608	0.073	[0.465–0.752]	0.1376
	MFR	3.70	0.16	0.495	0.077	[0.343–0.646]	0.9467
Women	BMI	20.90	0.27	0.596	0.086	[0.427–0.764]	0.2668
	FMI	4.17	0.32	0.614	0.092	[0.434–0.794]	0.2148
	SMI	8.96	0.24	0.579	0.095	[0.394–0.765]	0.4018
	MFR	1.67	0.29	0.628	0.098	[0.437–0.819]	0.1894

The diagnostic accuracy of the cut-off points for identifying injuries in men was the best for BMI (AUC = 0.623; Youden Index = 0.30) and SMI (AUC = 0.608; Youden Index = 0.23). The predictive potential for FMI was weaker (AUC = 0.572, Youden Index = 0.20). The results confirmed the inability to predict injuries based on MFR (AUC = 0.495, Youden Index = 0.16) (Table 2).

The results observed in women were opposite to the men. Indices based on fat mass component (FMI and MFR) showed greater predicting power than BMI and SMI (based on muscle mass component). The most accurate in predicting musculoskeletal injuries was MFR (AUC = 0.628; Youden = 0.29) and next FMI (AUC = 0.614; Youden = 0.32). The performance of BMI was slightly higher than SMI (AUC = 0.596, Youden Index = 0.27; AUC = 0.579, Youden Index = 0.24, respectively) (Table 2).

**Figure 1:**
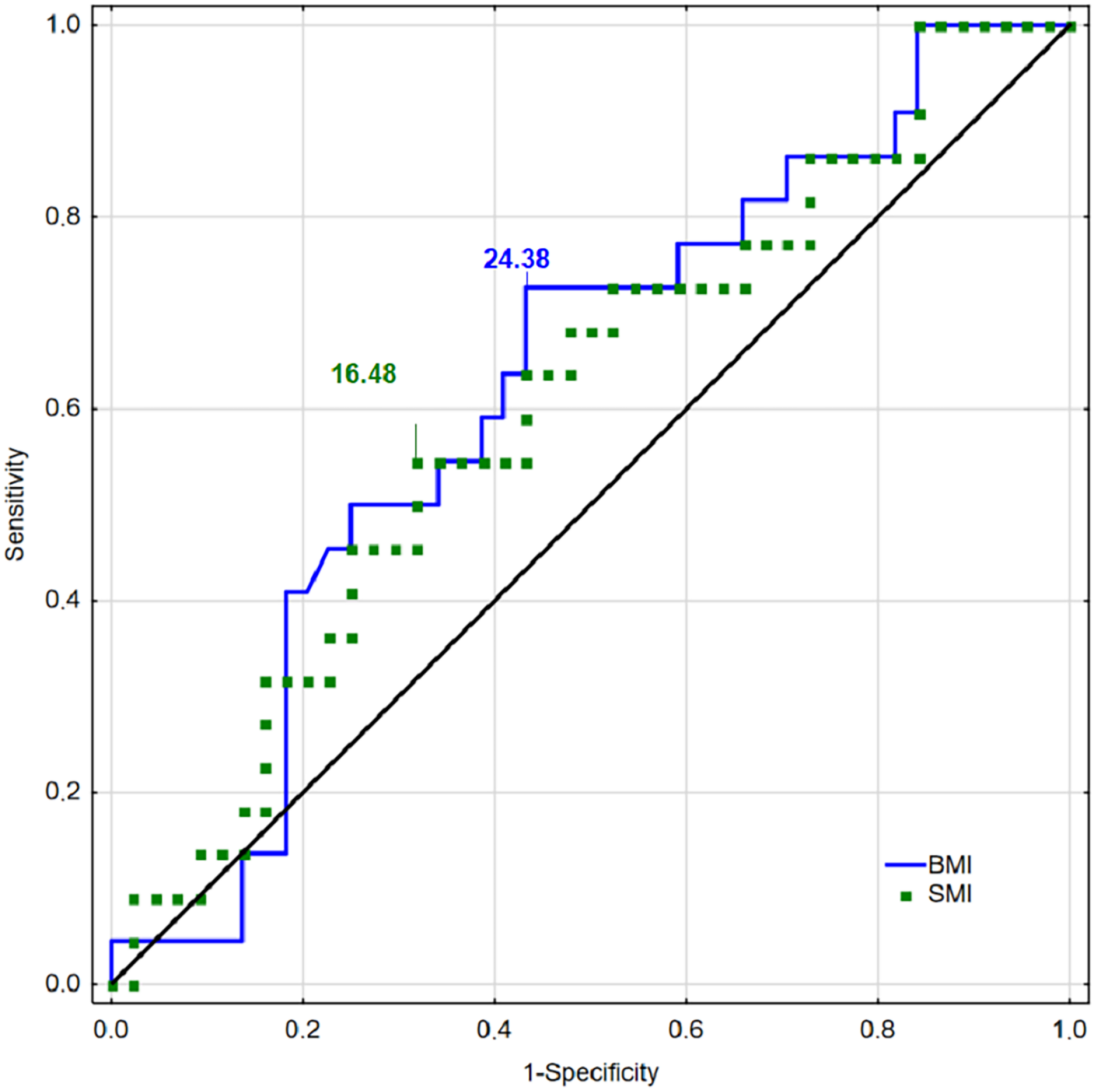
Receiver operating characteristic curves for BMI, FMI, SMI, and MFR for men.

**Figure 2:**
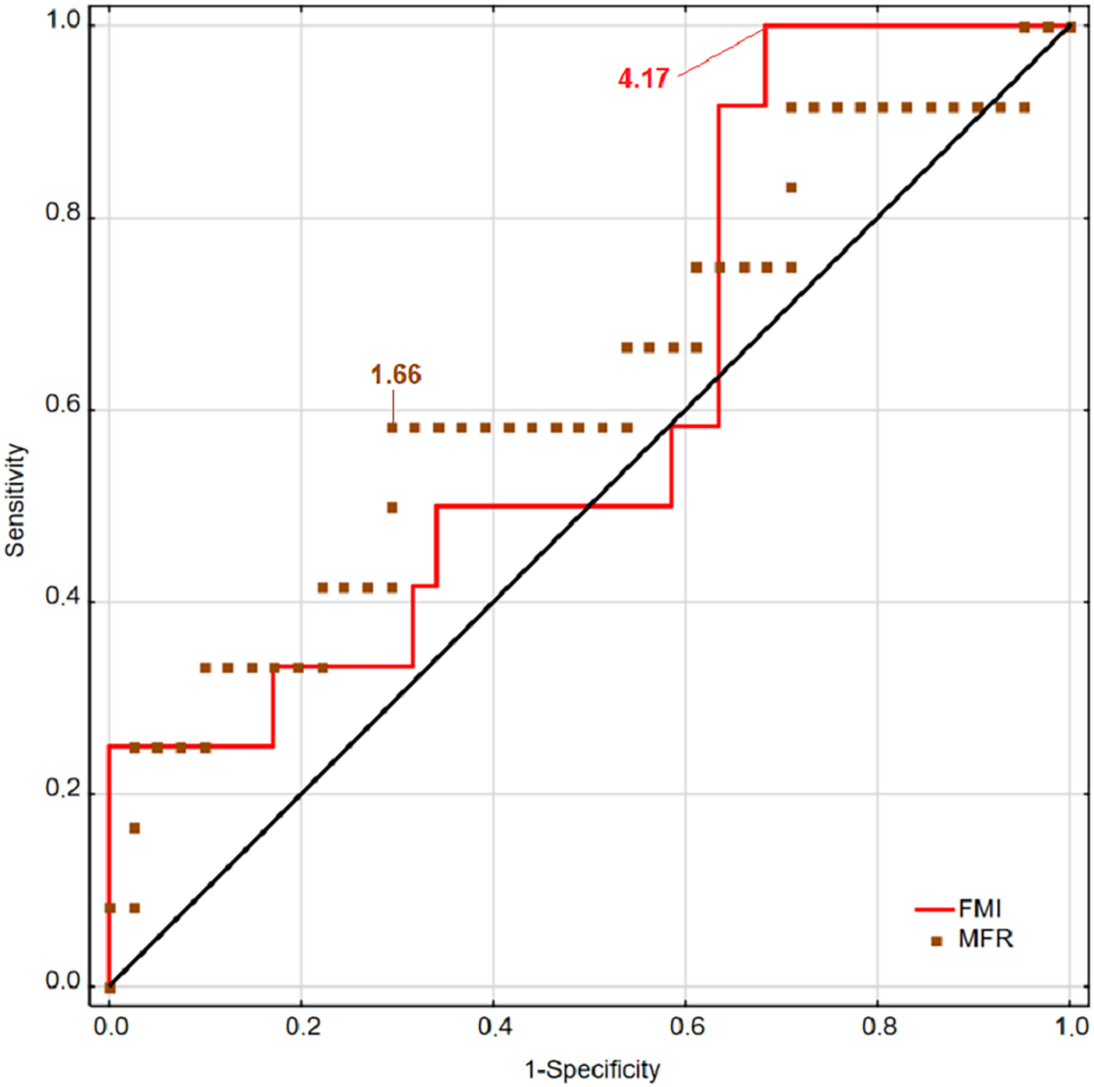
Receiver operating characteristic curves for BMI, FMI, SMI, and MFR for women.

ROC curves for BMI and SMI for men are presented in Fig. 1, and ROC curves for FMI and MFR are presented in Fig. 2. The presented curves showed the best values of sensitivity and specificity for injury detecting concern sexes.

## Discussion

This study indicated the cut-off points for body composition indices as cut-off points for higher risk of MI in healthy, physically active young adult men and women. Our results showed sex differences in performance of body mass component indices in detecting the risk of MI in men and women. The most identifying participants at risk of MI were BMI and SMI in men and MFR and FMI in women. Regarding the purpose of the work, conducted ROC analysis presented acceptable AUC suggesting that the resultant cut-off points were not due to chance (all AUC > 0.5) and effectively distinguished between healthy and injured participants (Havenetidis et al., 2017).

The mechanism of MI related to intrinsic factors could be low muscular strength, low muscular endurance, or low neuromuscular coordination (Taanila et al., 2015). Therefore, it seems that it could be the effect of too low muscle mass. Furthermore, the relationship between muscle mass, strength, and injury of the lower extremities may confirm it (Murphy, Connolly & Beynnon, 2003). Adequate skeletal muscle mass, especially in proper proportion to body fat (BF), guarantees strength and stability for the passive locomotor systems (Fukuoka et al., 2019). Conversely, an imbalance in those proportions may decrease the stability of joints and result in high injury risk; therefore, it is possible to hypothesize that various body composition indices help detect the risk of MI in physically active young adults. The incidence of MI during physical activity in physically active young adults remains high. The prevalence in the subgroup of men was higher (33.33%) than that of the women (22.64%) during the 12 months before the tests. In our young adults, the population reflects other results, that MI is a serious public health problem (Złotkowska et al., 2015). Our findings are consistent with results observed in military populations (Melloni et al., 2018; Mehri et al., 2010; Taanila et al., 2011; Havenetidis et al., 2017) and groups of athletes (Rosa et al., 2014; Dick, Agel & Marshall, 2007; Emery, 2003), and those who are active recreationally (Ezzat et al., 2016; Grimmer, Jones & Williams, 2000; McGuine, 2006; Rechel, Yard & Comstock, 2008). The prevalence varied from slightly less than 30% in military personnel to almost 50% of sports injuries in athletes. Rosa et al. (2014) reported even more frequent injury incidents in women (51%) than men (49.1%). Most of the injuries were severe during that time, with a recovery time longer than 10 days.

Apart from intrinsic injury risk factors, there is also a group of extrinsic risk factors independent of the injured person and related to many external variables (Taimela, Kujala & Osterman, 1990). These are the factors that contribute to the injury occurrence, such as abrupt increases in running mileage, switching from running on flat surfaces to hills, type of shoes, lack of variation in training programs and training methodology (van der Worp et al., 2015; Zaar et al., 2017; Sun et al., 2020). Extrinsic risk factors affect joint stability and muscle imbalance as well as intrinsic risk factors.

The ROC method indicated cut-off points body composition indices for detecting injury risk varied between men and women. However, calculated cut-off points for BMI and SMI for men and MFR and FMI for women showed high reliability due to high AUC (>0.5 and Youden index) and its possibility to injury risk estimation. The results are partly in line with other publications. A few showed that the resultant cut-off points could effectively distinguish injured and non-injured recruits based on BFP, FMI, and FFM, but not BMI (Havenetidis et al., 2017).

In some studies, the relationship between BMI and injury risk was bimodal for both sexes. The highest injury risk was in the groups of adults with the lowest BMI. In contrast, the risk of injury was lowest in the average BMI group, higher than the middle quantiles (the quantiles were calculated for the study population) (Jones et al., 2017). Another issue is that body size, which BMI represents, interacts with the level of physical fitness. Most aerobically fit, physically active adults are protected from injury regardless of their BMI level (Knapik et al., 2009). Overweight youths with a high level of physical activity were at increased risk of injury. However, even obese, active adults with a minimum, moderate physical fitness were better protected than healthy weight persons with low levels of physical fitness (Ezzat et al., 2016). Some interesting observations provide Hollander et al. (2020). A study among 73,640 soldiers was indicated higher injury risk with growing BMI.

We found no previous studies on physically active young adults that presented sensitivity, specificity, and cut-off points as predictors of injury concerning body composition indices. Therefore, our findings show new directions of scientific research and present new knowledge in physical activity research. Summarising this part of the discussion, we can conclude that other body composition indices are reliable for detecting injury risk among women.

The methodology of registering body composition varies. Some authors measured skinfolds and circumferences. There are simple skeletal muscle mass estimation formulas based on age, height, weight, and waist circumference (Heymsfield et al., 2020). In our studies, we used bioimpedance analysis (BIA), an accurate and reliable method for examining the relationship between body composition and injuries (Cowan et al., 2011; Havenetidis et al., 2017, Peake et al., 2012). These authors examined the usefulness of the body fat indices in detecting injuries in the military population. Regarding BFP and FMI, our results contradict Havenetidis et al. (2017) and Blacker et al. (2008), who observed relationships between BF indices and injuries. But our presented data were in accordance with Friedl et al. (2001) and a close agreement with results received with dual-energy X-ray absorptiometry (Kim et al., 2004). The authors showed that fat tissue measurements were not a significant risk factor for injury occurrence. In body composition indices (FMI and MFR), evaluated in our study, the adipose mass is the foundation for its formula. It could be possible that this variable influenced the results of those indices lowering its effectiveness in prognostic musculoskeletal injuries.

To our knowledge, there are not many studies that investigated body composition indices measure for the prognosis of injuries on average, physically active young adults. Chassé, Fergusson & Chen (2014) indicated an increased injury risk with higher BMI values among women but not among men. This observation is similar to the previously mentioned study by Hollander et al., 2020. In our study, we show BMI could be inappropriate for detecting injury among women but in men. The cause of this difference could be associated with the proportion of muscle to fat. On the other hand, Richmond, Kang & Emery (2013) confirmed that maintaining an appropriate body weight–height ratio is associated with lower injury risk. Several works show the values of SMI in healthy, young populations in different countries and continents, even with cut-off points for health and sarcopenia ranges, but there is no relation to injury mentioned (Alemán-Mateo & Ruiz Valenzuela, 2014; Schuna et al., 2015; Kim et al., 2004; Hwaung et al., 2019). The present study clearly shows that all indices predict injury factors, and their diagnostic performances are not the same. Different AUCs for each of them have confirmed this.

This study aimed to determine the cut-off points to classify participants as injured and healthy. The found cut-off points among men showed the best reliability for BMI = 24.38 and SMI = 16.40. Thus, the cut-off points for BMI and SMI were demonstrated as reliable in predicting injury prevalence for young, physically active men. Havenetidis et al. (2017), whose study analysed recruits, shows similar indices as a BMI = 25.3 (cut-off point = 22.24). Moreover, in the relevant research, the fat-free mass index (FFMI) was analysed, and the mean value was 21.54, with the cut-off point at 22.57. It is harder to compare this data for women due to the lack of similar analysis. But our observation showed that MFR = 1.67 and FMI = 4.17, indices highly tied with body fat, are appropriate for women to injury risk estimation. However, the observed differences mentioned above may be due to various study samples. Our study was conducted on physically active young adults concerning sexes, whereas a study by Havenetidis et al. (2017) was done on male recruits. Therefore, there is a need to explore the various body composition indices in injury risk prediction. Moreover, we postulate identifying cut-off points to each examined population to avoid interpretation errors.

There are limitations concerning data. Firstly, limited participants could have resulted in the lack of statistical significance of the designed ROC models. In addition, all indices are related to each other; BMI and BF interact with each other. So, this could lead to overestimating injury risk. Another shortcoming was the narrow range of age of 24 years. On the one hand, it was suitable for the low variability and the consistency of the group of men and women. However, on the other hand, the group did not represent the whole population of young adults, but only one cohort at the beginning of adulthood. Furthermore, there was a limitation in additional data on subjects’ physical conditioning assessment regarding potential fitness level differences that can influence injury occurrence.

## Conclusions

Men and women were injured equally often. This study suggests that body composition potentially contributes to injury risk as an intrinsic factor. Regarding the indices formulas, those based to a greater extent on muscle mass (BMI and SMI) have a potentially higher detection power of the risk of injuries among men. In contrast, those based on adipose tissue (FMI, MFR) are reliable indices for predicting musculoskeletal injury risk in physically active young women. The men with BMI over 24.38, an SMI over 16.40, and women with MFR over 1.67 and FMI over 4.17 are more likely to be injured. These body composition indices can be found for intrinsic factors related to injuries concerning sexes. Considering the possibility of intentional modifications of the proportions between body fat and muscles through diet, the chance of preventing injury is significant. The calculated cut-off points should be used with caution and should be confirmed in subsequent studies.

Our study shows new possibilities for injury risk estimation. Physical activity, besides the health benefits, relates to injury occurrence. Therefore, there is a need to decrease injury occurrence by identifying injury risk factors. Body composition is a fundamental factor of health. The knowledge of the usefulness of its indices in injury risk could help decrease injury risk.

However, future research should focus on (1) increasing the range of age of the participants among young adults instead of one age cohort, (2) extending the study to groups of various ages (children, youth, older adults) and athletes, (3) the verification of the calculated cut-off points for body mass indices in detecting the risk of injuries in prospective examinations, (4) developing research hypotheses that should be explored in the further studies.

## Supplemental Information

10.7717/peerj.12745/supp-1 Supplemental Information 1 Injury history questionnaire (English translation).

10.7717/peerj.12745/supp-2 Supplemental Information 2 Injury history questionnaire. Original form used in this study

10.7717/peerj.12745/supp-3 Supplemental Information 3 International Physical Activity Questionnaire (English translation).

10.7717/peerj.12745/supp-4 Supplemental Information 4 International Physical Activity Questionnaire (Polish).

10.7717/peerj.12745/supp-5 Supplemental Information 5 Data set.

## Additional Information and Declarations

### Data Availability

The following information was supplied regarding data availability: The raw measurements are available in the Supplemental File. The IHQ was developed by the authors and the IPAQ is open access:
https://sites.google.com/site/theipaq/

https://dapa-toolkit.mrc.ac.uk/instrument/53
