# Supplementary material for: The performance of body mass component indices in detecting risk of musculoskeletal injuries in physically active young men and women
Source: PeerJ. 2022 Jan 26;10:e12745. doi: 10.7717/peerj.12745 (PMC8800385; doi:10.7717/peerj.12745)
Supplement: Supplemental Information 1 [file peerj-10-12745-s001.pdf]

Injury History Questionnaire

Name: ....Surname: ....Birth date: ....

Injury definition - In these studies, the injury was defined as the occurrence of complaints during physical activity, which resulted in pain and discomfort in the locomotor system, causing temporary limitation or complete inability to continue physical activity.

1. Have you suffered any motor system injury between 01/10/2018 and 31/03/2019?

YES□*(**complete the table below)** NO □

2. Fill in the table below by entering the **NUMBER** of injuries in the cell corresponding to the part of the body that was injured during the period from 01/10/2018 to 31/03/2019

Body part	Injury number
Haed, neck, torso	
Upper limb -left	
Upper limb - right	
Lower limb - left	
Lower limb - right	
