# Supplementary material for: The performance of body mass component indices in detecting risk of musculoskeletal injuries in physically active young men and women
Source: PeerJ. 2022 Jan 26;10:e12745. doi: 10.7717/peerj.12745 (PMC8800385; doi:10.7717/peerj.12745)
Supplement: Supplemental Information 2 — Original form used in this study [file peerj-10-12745-s002.pdf]

Imię: ....Nazwisko: ....Data urodzenia: ....

1. Czy podejmujesz jakąkolwiek aktywność fizyczną? Tak □ Nie□

2. Czy posiadasz klasę sportowa? Tak □ Nie □

3. URAZ definiujemy jako powstanie dolegliwości w czasie podejmowanej aktywności fizycznej w wyniku, której doszło do pojawienia się bólu i/lub dyskomfortu w obrębie układu ruchu powodując czasowe ograniczenie lub zupełny brak możliwości kontynuowania aktywności fizycznej.

Czy doznałeś/doznałaś jakiegokolwiek urazu układu ruchu w okresie od 01.10.2017 do 01.10.2018.?

Tak □***(uzupełnij poniższą tabelę)** Nie □

4. Wypełnij poniższą tabelę wpisując **LICZBĘ** urazów w komórkę odpowiadającą części ciała, której dotyczył uraz odniesiony w okresie od 01.10.2017 do 01.10.2018.

Część ciała	Liczba urazów
Głowa, szyja, tułów	
Kończyna górna lewa	
Kończyna górna prawa	
Kończyna dolna lewa	
Kończyna dolna prawa	

5. Czy któryś z urazów został odniesiony w ciągu **ostatnich 6 tygodni** do dziś?

Tak □ Nie □
