# Supplementary material for: The performance of body mass component indices in detecting risk of musculoskeletal injuries in physically active young men and women
Source: PeerJ. 2022 Jan 26;10:e12745. doi: 10.7717/peerj.12745 (PMC8800385; doi:10.7717/peerj.12745)
Supplement: Supplemental Information 4 [file peerj-10-12745-s004.pdf]

Załącznik 2 Akieta IPAQ

IMIĘ................................ NAZWISKO...

Międzynarodowy Kwestionariusz Aktywności Fizycznej

Chciał(a)bym obecnie zadać kilka pytań dotyczących czasu spędzanego na czynnościach wymagających aktywności fizycznej. Dotyczą one wszystkich rodzajów aktywności fizycznej związane z życiem codziennym, z pracą i z wypoczynkiem. Pytania te zadajemy wszystkim, niezależnie od tego, czy ktoś uważa się za osobę aktywną fizycznie, czy też nie.

Pytania będą dotyczyć czynności związanych z aktywnością fizyczną w ciągu ostatnich 7 dni, tzn. od .... (podać dzień tygodnia) do wczoraj.

NAJPIERW JEDNAK CHCIAŁ(A)BYM ZAPYTAĆ, NA ILE OSTATNIE 7 DNI BYŁY TYPOWE, BIORĄC POD UWAGĘ NORMALNIE WYKONYWANE CZYNNOŚCI.

Czy w ciągu ostatnich 7 dni, tzn. od .... (podać dzień tygodnia) do wczoraj :

a. przez cały czas lub część czasu przebywał P. w szpitalu...	**Tak**	**Nie**
b. przez cały czas lub część czasu był P. chory..	**Tak**	**Nie**
c. przez cały czas lub część czasu odbywał P zajęcia rehabilitacyjne................................	**Tak**	**Nie**
d. przez cały czas lub część czasu przebywał P na urlopie..	**Tak**	**Nie**
e. jest P. w okresie rekonwalescencji po przebytej chorobie...	**Tak**	**Nie**
f. (tylko dla kobiet) jest P. w ciąży...	**Tak**	**Nie**

Proszę teraz pomyśleć o wszystkich czynnościach wykonywanych w ciągu ostatnich 7 dni w domu i w jego otoczeniu, w pracy zawodowej, związanych z przemieszczaniem się z miejsca na miejsce, np. drodze do pracy i z pracy, robieniu zakupów. Proszę także uwzględnić czynności wykonywane w czasie wolnym, tj. spacery, rekreacja, praca na działce, ćwiczenia fizyczne oraz sport. Najpierw zapytam P. o czynności wymagające dużego wysiłku fizycznego, następnie o czynności wymagające umiarkowanego, średniego wysiłku, a na koniec o spacery i inne czynności związane z chodzeniem oraz siedzeniem.

Na początek proszę przypomnieć sobie wszystkie czynności wymagające *intensywnego wysiłku fizycznego*, wykonywane w ciągu *ostatnich 7dni.*

Intensywny wysiłek fizyczny **wywołuje bardzo szybkie oddychanie i bardzo szybkie bicie serca**

Intensywnego wysiłku fizycznego wymaga np. dźwiganie ciężkich przedmiotów, kopanie ziemi, aerobik, szybki bieg, szybka jazda rowerem. Interesują nas tylko czynności, które trwały *co najmniej 10 min. bez przerwy.*

1. Czy w ciągu **ostatnich 7 dni** wykonywał/a P. czynności wymagające **intensywnego wysiłku fizycznego**?

   Tak – przez ile dni w ciągu ostatniego tygodnia? .... dni
   Nie (przejść do pyt. 3)
   Nie wiem/Nie jestem pewien(a) (przejść do pyt. 3)

2. Przeciętnie ile czasu wykonywał/a P. czynności wymagające **intensywnego wysiłku fizycznego** w ciągu takiego dnia?

   .... minut dziennie
   Nie wiem/Nie jestem pewien(a)

A teraz proszę przypomnieć sobie wszystkie czynności wymagające *umiarkowanego (średniego) wysiłku fizycznego* wykonywane w ciągu *ostatnich 7 dni.*

Umiarkowany wysiłek fizyczny **prowadzi do trochę szybszego oddychania i trochę szybszego bicia serca**

Umiarkowanego wysiłku fizycznego wymaga np. noszenie lżejszych ciężarów, jazda rowerem w normalnym tempie, gra w siatkówkę lub bardzo szybki marsz. Proszę jednak nie brać pod uwagę chodzenia. Chodzi znowu tylko czynności, które trwały *co najmniej 10 minut bez przerwy.*

3. Czy w ciągu **ostatnich 7 dni** wykonywał/a P. czynności wymagające umiarkowanego, średniego wysiłku fizycznego?

Tak – przez ile dni w ciągu ostatniego tygodnia? .... dni
Nie (przejść do pyt. 5)
Nie wiem/Nie jestem pewien(a) (przejść do pyt. 5)

4. Przeciętnie ile czasu wykonywał/a P. czynności wymagające **umiarkowanego wysiłku fizycznego** w ciągu takiego dnia?

.... minut dziennie
Nie wiem/Nie jestem pewien(a)

Teraz proszę przypomnieć sobie, ile czasu zajęło Panu/Pani *chodzenie* w ciągu *ostatnich 7 dni.* Interesuje nas chodzenie związane z pracą, chodzenie ulicą, np. po zakupy, do pracy, a także o spacery.
Chodzi znowu o chodzenie, które trwało *co najmniej 10 minut bez przerwy.*

5. Czy w ciągu **ostatnich 7 dni** chodził/a P. **co najmniej 10 min. bez przerwy**?

Tak – przez ile dni w ciągu ostatniego tygodnia? .... dni
Nie (przejść do pyt. 7)
Nie wiem/Nie jestem pewien(a) (przejść do pyt. 7)

6. Przeciętnie ile czasu poświęcał/a P. na **chodzenie lub spacery** w ciągu takiego dnia?

.... minut dziennie
Nie wiem/Nie jestem pewien(a)

A ile czasu w ostatnim tygodniu spędzał Pan/Pani *siedząc*? Tym razem *proszę uwzględnić tylko dni powszednie*, tzn. proszę pominąć sobotę i niedzielę. Chodzi np. o siedzenie przy biurku, siedzenie podczas odwiedzin u znajomych, podczas czytania, a także siedzenie lub leżenie podczas oglądania telewizji. Proszę uwzględnić czas spędzony na siedzeniu w domu, w pracy, w szkole, w pojazdach i w innych miejscach.

7. Biorąc pod uwagę **dni powszednie w ciągu ostatniego tygodnia**, ile zazwyczaj czasu w ciągu dnia spędzał/a P. **siedząc**?

.... minut dziennie
Nie wiem/Nie jestem pewien(a)
